# In Silico Evaluation of the Antimicrobial Activity of Thymol—Major Compounds in the Essential Oil of *Lippia thymoides* Mart. & Schauer (Verbenaceae)

**DOI:** 10.3390/molecules27154768

**Published:** 2022-07-26

**Authors:** Jorddy Neves Cruz, Sebastião Gomes Silva, Daniel Santiago Pereira, Antônio Pedro da Silva Souza Filho, Mozaniel Santana de Oliveira, Rafael Rodrigues Lima, Eloisa Helena de Aguiar Andrade

**Affiliations:** 1 Laboratory of Functional and Structural Biology, Institute of Biological Sciences, Federal University of Pará, Belém 66075-110, PA, Brazil; rafalima@ufpa.br; 2 Adolpho Ducke Laboratory, Museu Paraense Emílio Goeldi, Belém 66077-830, PA, Brazil; profsebastiaogs@gmail.com (S.G.S.); mozaniel.oliveira@yahoo.com.br (M.S.d.O.); eloisa@museu-goeldi.br (E.H.d.A.A.); 3 Brazilian Agricultural Research Corporation (EMBRAPA), Belém 66095-100, PA, Brazil; daniel.pereira@embrapa.br (D.S.P.); antonio-pedro.filho@embrapa.br (A.P.d.S.S.F.)

**Keywords:** molecular modeling, natural products, biological activity, interaction mechanism

## Abstract

In this paper, we evaluated the drug-receptor interactions responsible for the antimicrobial activity of thymol, the major compound present in the essential oil (EO) of *Lippia thymoides (L. thymoides)* Mart. & Schauer (Verbenaceae). It was previously reported that this EO exhibits antimicrobial activity against *Candida albicans* (*C. albicans*), *Staphylococcus aureus* (*S. aureus*), and *Escherichia coli* (*E. coli*). Therefore, we used molecular docking, molecular dynamics simulations, and free energy calculations to investigate the interaction of thymol with pharmacological receptors of interest to combat these pathogens. We found that thymol interacted favorably with the active sites of the microorganisms’ molecular targets. MolDock Score results for systems formed with CYP51 (*C. albicans*), Dihydrofolate reductase (*S. aureus*), and Dihydropteroate synthase (*E. coli*) were −77.85, −67.53, and −60.88, respectively. Throughout the duration of the MD simulations, thymol continued interacting with the binding pocket of the molecular target of each microorganism. The van der Waals (ΔE_vdW_ = −24.88, −26.44, −21.71 kcal/mol, respectively) and electrostatic interaction energies (ΔE_ele_ = −3.94, −11.07, −12.43 kcal/mol, respectively) and the nonpolar solvation energies (ΔG_NP_ = −3.37, −3.25, −2.93 kcal/mol, respectively) were mainly responsible for the formation of complexes with CYP51 (*C. albicans*), Dihydrofolate reductase (*S. aureus*), and Dihydropteroate synthase (*E. coli*).

## 1. Introduction

Essential oils (EOs), formed as secondary metabolites of aromatic plants, are biosynthesized in different plant organs such as flowers, leaves, and stems, among others [1,2]. These EOs chemical compositions and yields can change due to natural factors such as physiological, environmental, geographic, genetic, and plant evolution [3,4]. These oils play essential roles in plant protection and communication [5,6].

In the industry, EOs have been widely studied, mainly because of their potential applications as antimicrobials [7]. Over the years, the volatile compounds present in them have been employed for several pharmacological activities, such as antioxidant, anticancer, antiprotozoal, antimicrobial, anti-inflammatory, phytotoxic, and neuroprotective activities [8,9,10,11,12]. In a recent study, Tanrıkulu et al. [13] demonstrated that species such as *Ocimum basilicum* and *Thymbra spicata* show good antioxidant and antimicrobial activities against *Staphylococcus aureus*, *Streptomyces murinus*, *Micrococcus luteus*, *Bacillus subtilis*, *Klebsiella pneumoniae*, *Pseudomonas aeruginosa*, *Yersina enterocolitica*, *Proteus algilus vulgaris*, and *Candida algilis*, demonstrating the importance of EOs in the industry.

Although the antimicrobial action of EOs is not yet fully understood, it can be attributed to their ability to permeate the cell wall of microorganisms, which arises from their diverse and synergistic chemical compositions. The hydrophobic nature of EOs enables the partitioning of lipids from the cell membrane and mitochondria, making them more permeable; consequently, ions and critical cellular components (lipids, proteins, and nucleic acids) are extravasated from the cells, leading to eventual cell death. Generally, EOs have more action on gram-negative than on gram-positive bacterium, owing to the hydrophobic components of these oils interacting with the cell membranes of the former [14,15,16].

Different methods have been used to assess the antibacterial and antifungal properties of EOs. The most commonly used methods are the agar disk diffusion, minimum inhibition concentration (MIC), minimum bacterial concentration (MBC), and minimum fungicidal concentration (MFC) methods. Because the agar disk diffusion method is limited by the hydrophobic nature of EOs and plant extracts, preventing their uniform diffusion through the agar medium, most authors report their obtained results via the MIC, MBC, and MFC methods [17].

**Figure 1:**
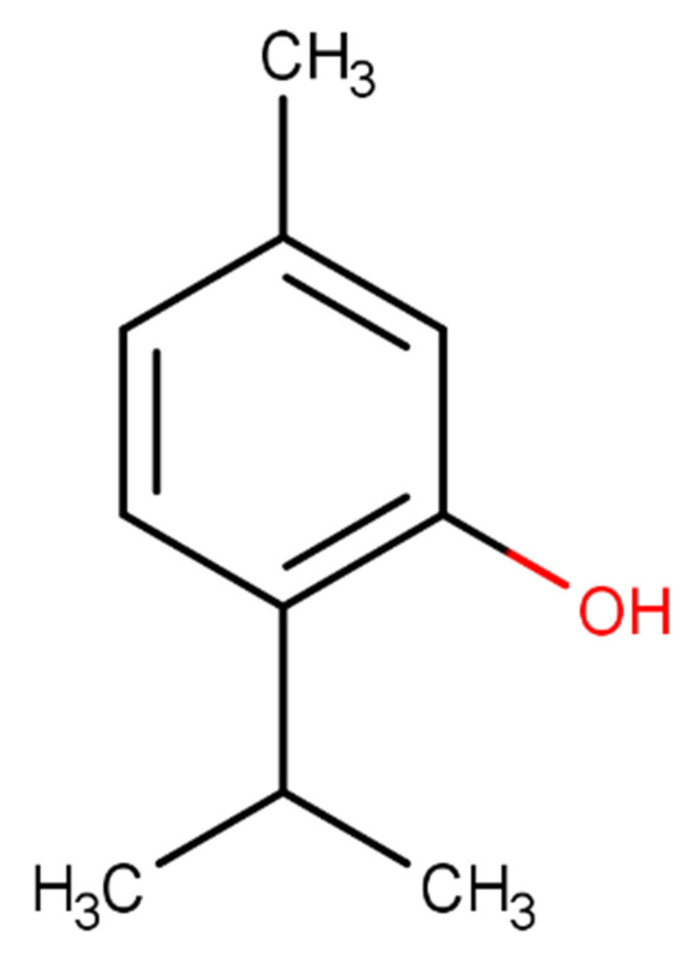
The molecular structure of thymol.

Silva et al. [18] evaluated the antimicrobial activity of EOs from *Lippia thymoides* Mart. & Schauer (Verbenaceae) against *C. albicans*, *S. aureus*, and *E. coli*. In this study, we used molecular modeling approaches to further analyze the investigations performed by Silva et al. We decided to conduct these investigations in silico to deepen our understanding of the interactions of volatile compounds with molecular targets that are vital for the viability of these microorganisms. Previous publications have shown that these approaches successfully reveal how drug-receptor interactions occur [19,20,21,22]. Therefore, we used molecular docking, molecular dynamics (MD) simulations, and affinity energy calculations to investigate how thymol (Figure 1)—the major compound of the EO from *L. thymoides*—interacts with the molecular targets from *C. albicans*, *S. aureus*, and *E. coli*.

## 2. Results and Discussions

### 2.1. Molecular Binding Mode

According to the molecular docking results, the thymol interacted favorably with the binding sites of the proteins (Table 1).

**Figure 2:**
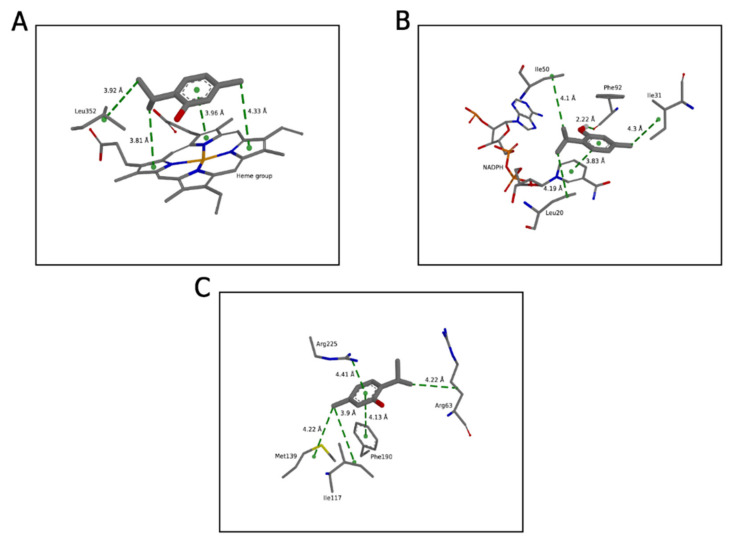
Intermolecular interactions of the drug-receptor systems. Molecular binding mode of thymol interacting with the active site residues of (**A**) CYP51 (*C. albicans*), (**B**) dihydrofolate reductase (*S. aureus*), and (**C**) dihydropteroate synthase (*E. coli*).

We identified the residues with which the ligand interacted and the chemical nature of these interactions (Figure 2).

**Figure 3:**
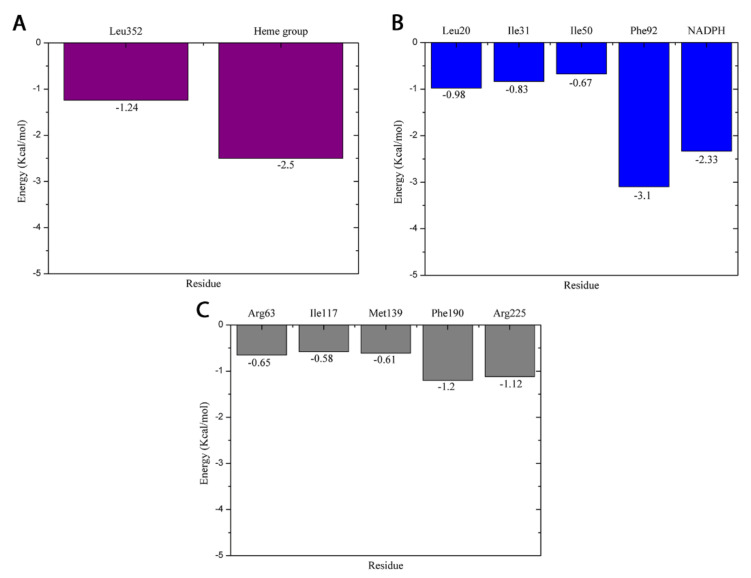
Per-residue free energy decomposition: (**A**) CYP51 (*C. albicans*), (**B**) dihydrofolate reductase (*S. aureus*), and (**C**) dihydropteroate synthase (*E. coli*).

After molecular docking, we performed MD simulations of 100 ns to evaluate how thymol can interact with the active site of the molecular targets. We then performed per-residue free energy decomposition using the MM/GBSA approach to evaluate the energetic contribution of the residues to the formation of the receptor-ligand complex, as shown in Figure 3.

EOs target the membrane of microorganisms such as *C. albicans*, *S. aureus*, and *E. coli* [23]. The total or partial rupture of the membrane allows the extravasation of intracellular liquid, causing a hydroelectrolytic imbalance capable of causing the death of the microorganism [24]. In addition, after passage through the membrane, EO compounds are free to interact with molecular targets essential for bacterial and fungal viability [25]. Thymol, for example, in our results, was shown to be able to interact with CYP51, Dihydrofolate reductase, and dihydropteroate synthase proteins that are critical for parasite viability. But in addition to these molecular targets, there are reports that this same compound can interact with other proteins. Dutta et al. concluded that the thymol in the EO of Trachyspermum ammi interacted with the pocket binding of glucosamine-6-phosphate synthase. The observed molecular interactions were essentially hydrophobic [26]. Some authors have identified that thymol can interact with dihydro-folate reductase from *S. aureus* [27,28]. Barbosa et al. (2021) determined that thymol can also inhibit NorA efflux pump inhibition in multidrug-resistant (MDR) *S. aureus* strains. In this paper, the authors evaluated that thymol establishes hydrophobic, hydrophilic, and electrostatic molecular interactions in the binding site [29]. Nagle et al. used thymol as a scaffold to perform a computer-aided drug design. Thymol and analogous compounds were investigated for their antimicrobial properties against *E. coli* and *S. aureus*. The molecular target chosen for the in silico studies was glucosamine-6-phosphate synthase GlcN-6-P synthase. The docking results showed that thymol and analogous showed a favorable interaction with the active site of the protein and their interactions were hydrophobic and electrostatic [30].

At the active site of CYP51 (*C. albicans*), the thymol established interactions with the heme group and Leu352. The interactions with the heme group were hydrophobic; one was of the π-π type and the other two were of the π-alkyl type. In addition to these three interactions, the heme group participated in the binding of the compound with the binding pocket, contributing with an energy value of −2.5 Kcal/mol. With Leu352, the interaction was of the alkyl type, and throughout the simulations, this residue provided an energy value of −1.24 Kcal/mol.

In the binding pocket of dihydrofolate reductase (*S. aureus*), thymol established a hydrogen bond with Phe92, and the energy value of its interaction was −3.1 Kcal/mol. Alkyl hydrophobic interactions were formed with Ile20, Ile31, and Ile50 with energy contributions of −0.98, −0.83, −0.67 Kcal/mol, respectively. The docking simulations demonstrated that NAPH established π-π interaction with thymol reaching energy value of −2.33 Kcal/mol throughout the MD simulations.

At the active site of dihydropteroate synthase (*E. coli*), the ligand formed alkyl type hydrophobic interactions with Arg63, Ile117, and Met139 with energy contribution values for a compost binding of −0.65, −0.58 e 0.61 Kcal/mol, respectively. With Arg225, the interaction occurred with energy value of −1.12 and a π-π interaction was established between the benzene ring and the guanidino group of the amino acid. Another π–π interaction was formed between the thymol and Phe190 side chain, exhibiting an energy value of −1.2 Kcal/mol.

### 2.2. DM Trajectory Stability

**Figure 4:**
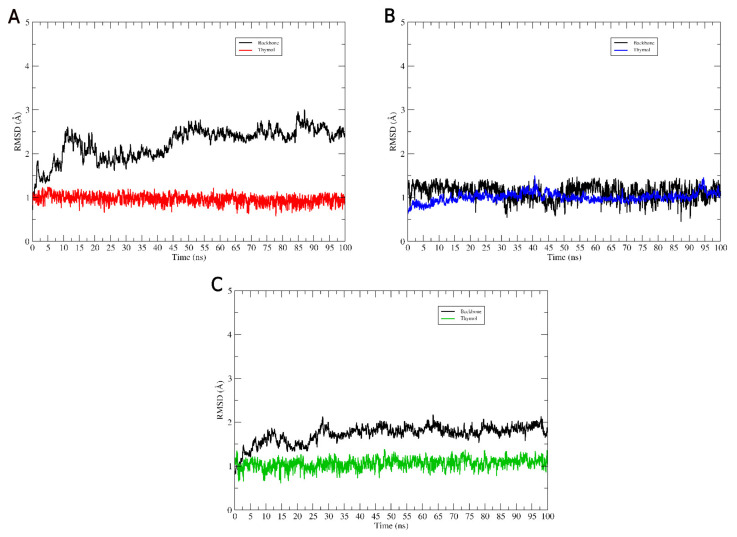
Intermolecular interactions of the drug-receptor systems. Molecular binding mode of thymol interacting with the active site residues of (**A**) CYP51 (*C. albicans*), (**B**) dihydrofolate reductase (*S. aureus*), and (**C**) dihydropteroate synthase (*E. coli*).

We used root-mean-square deviation (RMSD) calculations to assess each protein’s structural changes in the ligand and backbone. The RMSD of the ligand was calculated using its heavy atoms, while the Cα atoms were used for the RMSD plot of the protein backbone (Figure 4).

The RMSD values of the ligands in all systems showed minor variations along the trajectories. Thus, the ligand was accommodated in its binding site, preserving its interactions with the catalytic residues. The three-dimensional structures of the proteins did not undergo any change that would compromise the maintenance of the systems formed with thymol. Throughout the entire trajectory, the inhibitors remained in interaction with the proteins.

### 2.3. Free Energy Calculation of the Thymol-Receptor Complexes

In this study, the free energies of the thymol-receptor complexes were calculated to assess whether thymol interacts favorably with the target protein of each microorganism. For this, we used the MM/GBSA method, and the free energy and its energetic components were calculated using the last 500 frames of the MD trajectories. The results are presented in Table 2.

The main contributions to the formation of the complexes were the van der Waals (ΔE_vdW_), electrostatic (ΔE_electrostatic_), and nonpolar solvation (ΔG_nonpol_) energies. The negative values of ΔG_bind_ demonstrated that the interaction of the ligand with these molecular targets is favorable. Based on all our results, we can state that thymol is capable of interacting with and thereby inhibiting these molecular targets.

## 3. Materials and Methods

### 3.1. Molecular Docking

For the molecular docking study, thymol was selected since it was the major compound from the essential oil isolated from the leaves of *L. thymoides* Mart. & Schauer (Verbenaceae). Molecular docking was used to investigate the interaction between thymol and essential proteins of *C. albicans*, *S. aureus,* and *E. coli*. The proteins used as a molecular target are essential for the metabolic pathways of such microorganisms, in addition to being reported in the literature as targets for natural and synthetic products that combat these pathogens [31,32,33,34].

Thymol (2-Isopropyl-5-methylphenol) is a volatile substance present in the essential oil of several species. This substance is a monoterpenoid belongs being derived from a hydride of a p-cymene [35]. Thymol was drawn in GaussView 6 [36], and its structure was optimized via B3LYP/6-31G * [37] using the Gaussian quantum chemistry software 16 [38]. The three-dimensional structures of the proteins used as molecular targets were obtained from the Protein Data Bank (www.rcsb.org (1 January 2022). The corresponding PDB IDs are 5V5Z (*C. albicans*—SC5314) [39], 2W9H (*S. aureus*—ATCC) [33], and 1AJ2 (*E. coli*—ATCC) [34]. To study the interaction mode of this molecule with target proteins for drug action, the software Molegro Virtual Docker 5.5 [40]. The MolDock Score (GRID) function was used with a Grid resolution of 0.30 Å and radius of 7 Å, encompassing the entire crystallographic ligand-binding cavity found in the PDB of each protein. The MolDock SE algorithm was used with the number of runs equal to 10; 1500 max interactions, and max population size equal to 50. The full evaluation of 300 steps with neighbor distance factor equal to 1 and energy threshold equal to 100 was used during the molecular docking simulation. The RMSD limit for multiple cluster poses was set to <1.00 Å.

### 3.2. MD Simulations

The charges of the thymol atoms were calculated using the restrained electrostatic potential protocol using HF/6-31G * [41]. Parameter files were built using the Antechamber and General Amber Force Field [42]. The protonation states of the protein residues were studied from the results obtained using the PROPKA server [43].

The proteins were described by the ff14SB force field [44] in all simulations, with explicit water molecules described by the TIP3P model [45]. Each system was solvated in an octahedron periodic box with a 12 Å cutting radius in all directions from the solute. An adequate number of counterions were added to neutralize the partial charge of the systems.

The MD simulations were performed using the Amber 16 software [46,47]. Energy minimizations were performed with the sander module, while the heating, balance and production steps were performed with pmemd. CUDA.

The system’s energy minimization took place in three steps. In the first stage, 2000 cycles were executed using the steepest descent method and conjugate gradient algorithms, applying a harmonic force constant of 50 kcal·mol^−1^·Å^−2^ about the solute. In the second stage, the harmonic force constant applied to the solute was 25 kcal·mol^−1^·Å^−2^, and 1000 more cycles were run using the steepest descent method and conjugate gradient algorithms. In the last step, the constraints were removed, and 1000 cycles were run using the steepest descent method and conjugate gradient algorithm.

900 ps simulations were run to increase the system temperature from 0 to 300 k. Warming up was carried out in three steps. In the first, the solute was constrained with a harmonic force constant of 25 kcal·mol^−1^·Å^−2^, in this way, only the solvent and counterions were free to move. In the next two steps, the harmonic force constant was removed. To balance the complexes, we run 2 ns simulations at constant temperature and without restrictions. Then, for each complex, 100 ns of MD simulation with NVT ensemble were generated.

The particle mesh Ewald method [48] was used for calculating the electrostatic interaction energies, and the bonds involving hydrogen atoms were restricted with the SHAKE algorithm [49]. The temperature was controlled using a Langevin thermostat [50] with a collision frequency of 2 ps^−1^.

### 3.3. Free Energy Calculations

The free energy calculations were performed using the molecular mechanic’s generalized boron surface area (MM/GBSA) method [51,52,53]. For these calculations, we used the last 5 ns of the MD simulation trajectories. The free energy was calculated as follows:

ΔG_bind_ = ΔH − TΔS ≈ ΔE_MM_ + ΔG_solv_ − TΔS (1)

where ΔG_bind_ is the free energy of the complex, resulting from the sum of the molecular mechanics energy (ΔE_MM_), desolvation free energy (ΔG_solv_), and entropy (−TΔS).

ΔE_MM_ = ΔE_internal_ + ΔE_electrostatic_ + ΔE_vdW_ (2)

The energy of molecular gas phase mechanics (ΔEMM) can be described by the sum of the internal energy contributions (ΔE_internal_); sum of the connection, angle, and dihedral energies; electrostatic contributions (ΔE_electrostatic_); and van der Waals terms (ΔE_vdW_).

ΔG_solv_ = ΔG_GB_ + ΔG_nonpol_ (3)

The desolvation free energy (ΔG_solv_) is the sum of the polar (ΔG_GB_) and nonpolar (ΔG_nonpol_) contributions. The polar desolvation term was calculated using the implicit generalized born (GB) approach.

## 4. Conclusions

According to our results for the binding energies obtained using the MM/GBSA method, thymol interacts favorably with the molecular targets of microorganisms. The binding free energies (ΔG_bind_) for thymol interacting with CYP51, dihydrofolate reductase, and dihydropteroate synthase proteins demonstrate that the formation of the complexes is favorable; the ΔG_bind_ values obtained were: −20.04, −24.73, −17.84 kcal/mol, respectively. During the 100 ns of MD simulations, thymol remained in interaction with the binding pockets of the enzymes. The RMSD values obtained over 100 ns of MD simulation showed that the thymol are stable in the binding pocket. The main interactions established by the ligand with the active site residues were found to be hydrophobic.

## Figures and Tables

**Table 1:** Scoring functions obtained with the MolDock score.

Drug Target	MolDock Score (Kcal/mol)
CYP51 (*C. albicans*)	−77.85
Dihydrofolate reductase (*S. aureus*)	−67.53
Dihydropteroate synthase (*E. coli*)	−60.88

**Table 2:** Binding energy values (ΔG_bind_) of the drug-receptor systems. ΔE_vdW_, van der Waals contributions; ΔE_electrostatic_, electrostatic energy; ΔG_GB_, polar solvation energy; ΔG_nonpol_, nonpolar solvation energy. All values are in kcal/mol.

System	ΔE_vdW_	ΔE_ele_	ΔG_GB_	ΔG_NP_	ΔG_bind_
CYP51(*C. albicans*)	−24.88	−3.94	12.15	−3.37	−20.04
dihydrofolate reductase (*S. aureus*)	−26.44	−11.07	16.03	−3.25	−24.73
dihydropteroate synthase (*E. coli*)	−21.71	−12.43	19.23	−2.93	−17.84

## Data Availability

Not applicable.

## Sample Availability

Samples of the *L. thymoides* Mart. & Schauer (Verbenaceae) are available from the Museu Emilio Goeldi.
